# Nasal high flow treatment in preterm infants

**DOI:** 10.1186/s40748-017-0056-y

**Published:** 2017-09-06

**Authors:** Calum T. Roberts, Kate A. Hodgson

**Affiliations:** 1 Newborn Research Centre, The Royal Women’s Hospital, Locked Bag 300, Flemington Road, Parkville 3052, Melbourne, VIC Australia; 2 Department of Obstetrics and Gynaecology, The University of Melbourne, Melbourne, Australia

**Keywords:** Nasal high flow, Continuous positive airway pressure, Non-invasive ventilation, Infant, premature

## Abstract

Nasal High Flow (HF) is a mode of ‘non-invasive’ respiratory support for preterm infants, with several potential modes of action, including generation of distending airway pressure, washout of the nasopharyngeal dead space, reduction of work of breathing, and heating and humidification of inspired gas. HF has several potential advantages over continuous positive airway pressure (CPAP), the most commonly applied form of non-invasive support, such as reduced nasal trauma, ease of use, and infant comfort, which has led to its rapid adoption into neonatal care. In recent years, HF has become a well-established and commonly applied treatment in neonatal care.

Recent trials comparing HF and CPAP as primary support have had differing results. Meta-analyses suggest that primary HF results in an increased risk of treatment failure, but that ‘rescue’ CPAP use in those infants with HF failure results in no greater risk of mechanical ventilation. Even in studies with higher rates of HF failure, the majority of infants were successfully treated with HF, and rates of important neonatal morbidities did not differ between treatment groups. Importantly, these studies have included only infants born at ≥28 weeks’ gestational age (GA). The decision whether to apply primary HF will depend on the value placed on its advantages over CPAP by clinicians, the approach to surfactant treatment, and the severity of respiratory disease in the relevant population of preterm infants.

Post-extubation HF use results in similar rates of treatment failure, mechanical ventilation, and adverse events compared to CPAP. Post-extubation HF appears most suited to infants ≥28 weeks; there are few published data for infants below this gestation, and available evidence suggests that these infants are at high risk of HF failure, although rates of intubation and other morbidities are similar to those seen with CPAP. There is no evidence that using HF to ‘wean’ off CPAP allows for respiratory support to be ceased more quickly, but given its advantages it would appear to be a suitable alternative in infants who require ongoing non-invasive support. Safety data from randomised trials are reassuring, although more evidence in extremely preterm infants (<28 weeks’ GA) is required.

## Background

Respiratory distress syndrome (RDS) and sequelae such as bronchopulmonary dysplasia (BPD) are substantial contributors to adverse outcomes in preterm infants. The use of ‘non-invasive’ respiratory support (without an endotracheal tube) is one strategy clinicians have adopted to improve management of these conditions. For several decades, nasal continuous positive airway pressure (CPAP) has been the principal mode of non-invasive respiratory support for preterm infants. CPAP is effective as post-extubation support, [1] and as primary respiratory support (the first respiratory support applied after initial stabilisation). CPAP is a feasible and effective alternative to routine intubation and ventilation after birth, even for those infants born at <28 weeks’ gestational age (GA) [2–5]. The use of CPAP as primary respiratory support may also improve long-term outcomes such as death or BPD, [6] and is recommended in current consensus guidelines for preterm infants with RDS [7].

While effective, CPAP does have some disadvantages: it requires the use of bulky interfaces, must be managed by highly skilled nursing staff to be effective, and has been associated with adverse outcomes, such as nasal trauma [8] and pneumothorax [3]. There is also significant heterogeneity of CPAP devices and interfaces, which may influence efficacy in research and clinical settings [9].

**Fig. 1:**
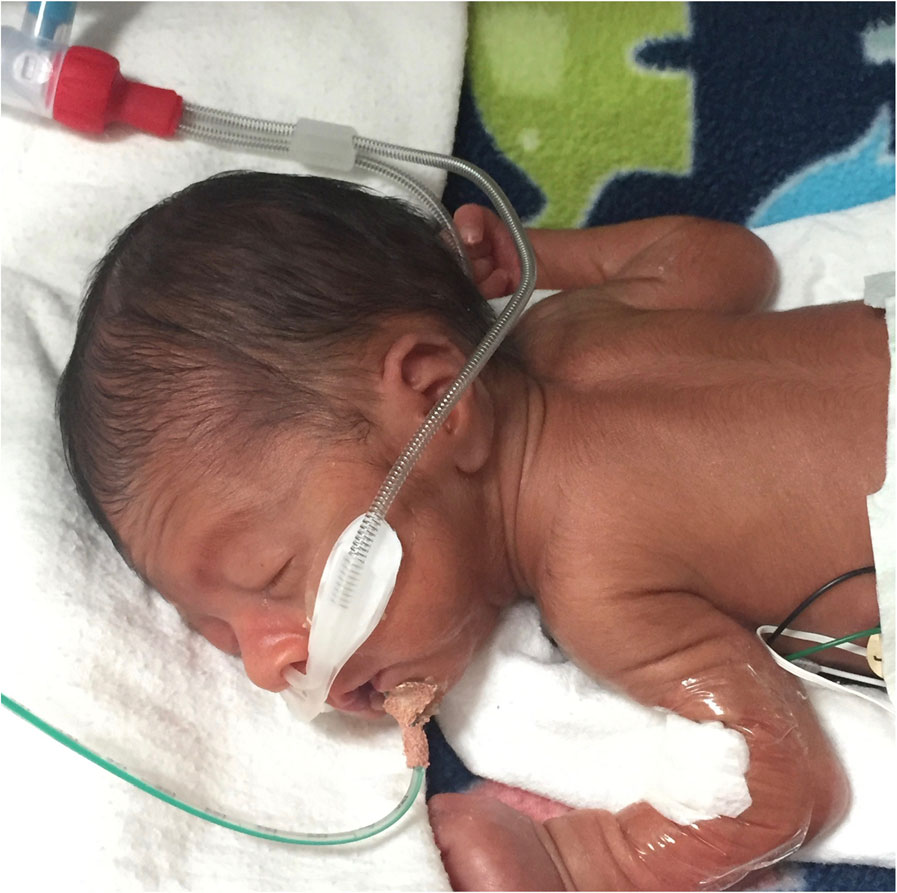
A preterm infant receiving High Flow support

Interest in nasal High Flow (HF), an alternative mode of non-invasive respiratory support, has grown dramatically in recent years. HF treatment refers to the delivery of heated, humidified, blended air and oxygen, via small loose-fitting nasal cannulae (Fig. 1), at >1 Litre per minute (L/min) [10]. Initially HF was applied clinically without good quality evidence of efficacy and safety. However, the publication of several recent large randomised clinical trials has allowed clinicians to make more informed decisions about which infants can appropriately receive HF treatment [11–14]

.

## Mechanisms of action

Several mechanisms of action for HF have been proposed, which can be summarised as follows: generation of distending pressure; ‘washout’ of the nasopharyngeal dead space; provision of gas flow sufficient to reduce inspiratory resistance and work of breathing; and provision of adequate gas conditioning [15].

### Distending pressure

CPAP systems generate a continuous distending pressure, which is set and usually measured. In comparison, HF devices use a set gas flow, but do not target, or measure, the applied pressure. Early clinical studies of HF in neonates focused on measuring the distending pressure resulting from HF therapy, both as a proposed mechanism of action, and due to concern that application of an uncontrolled, potentially high pressure may lead to pulmonary over-distension. Studies have identified three key factors that result in increased distending pressure during HF treatment: increasing gas flow, increased ratio of the diameter of the cannulae to that of the nares, and lower infant weight [16–18]. Appropriate sizing of the cannulae, such that there is a gas ‘leak’ around them in the nares, is an important aspect of applying HF safely. Studies in which the prongs are sized according to these recommendations have found that within the flow range currently applied in neonates (up to 8 L/min), upper airway pressures are similar to those routinely used with CPAP [19–21].

### Dead space washout

The anatomical dead space is the volume of gas between external nares and terminal bronchioles, which usually has gas concentrations similar to the alveoli (i.e. higher carbon dioxide and lower oxygen concentrations than atmospheric air) [22]. In theory, the ‘wash out’ of the nasopharyngeal gas space by HF may reduce anatomical dead space and improve gas exchange, although data supporting this concept come mostly from lab and animal studies [15, 23]. A recent benchtop study, using a neonatal model with simulated spontaneous breathing, found that with the mouth closed, carbon dioxide washout times were significantly shorter with HF than with CPAP, although the two modes did not differ with the mouth open. The authors noted that this effect might be particularly important in more preterm infants, who have a high dead space-to-tidal volume ratio [24]. There are no in vivo data specifically supporting this mechanism of action from studies of neonatal patients.

### Work of breathing

The nasopharynx has a relatively large surface area with resulting resistance to gas flow, which might be minimised by the use of HF to provide a gas flow at or above the peak inspiratory flow of the patient [15]. Physiological studies, using measures such as respiratory inductance plethysmography and diaphragmatic electrical activity, have generally supported the concept that work of breathing can be reduced by HF treatment. Results of comparisons with CPAP however have been conflicting, with some studies finding HF to be of similar efficacy, [25–27] and others finding CPAP more effective [28, 29].

### Gas conditioning

Delivery of unconditioned gases can have a number of adverse effects on respiratory function, including impaired mucociliary function and thickening of secretions, damage to the airway mucosa, [30–32] and a reduction in pulmonary compliance and functional residual capacity [33]. Furthermore, heating and humidification of environmental gas by the upper airway mucosa is associated with both caloric and evaporative heat loss, [34] and can result in hypothermia, factors that are all specifically important to preterm infants [35–37]. Therefore, appropriate gas conditioning has been postulated as a potential benefit resulting from HF treatment [15]. Lab studies have found HF devices to produce absolute humidity levels similar to, or slightly below, those achieved by CPAP devices [38, 39]. Whether these differences are clinically significant is unclear.

## Advantages of high flow

The perception that HF has several advantages over CPAP, and is a ‘less invasive’ mode of therapy, was a major contributory factor in the growth of its use in neonatal units. In surveys conducted in Australia and the UK, cited benefits included reduced nasal trauma, increased infant comfort, easier access for parental feeding and skin-to-skin care, and ease of set-up and use for staff [40–42]. Over time, evidence has accumulated to support the veracity of many of these perceived benefits.

The majority of HF randomised controlled trials (RCTs) have shown a reduction in nasal trauma with HF in comparison with CPAP, [11, 12, 14, 43, 44] although two showed no difference in trauma rates [45, 46]. The Cochrane Review included pooled analyses of several of these studies: as post-extubation treatment HF was associated with a significant reduction in nasal trauma versus CPAP (RR 0.64, 95% confidence interval [CI] 0.51, 0.79), but as primary respiratory support the difference was not significant (RR 0.62, 95% CI 0.34, 1.15) [10].

Of two studies to assess patient comfort using validated scales, one showed no difference in pain score between HF and CPAP, [47] whereas a second found HF infants to have significantly lower pain scores and salivary cortisol levels [48].

Another perceived advantage is that HF better enables oral feeding, specifically for infants attempting to breast-feed. However, neither individual RCTs, nor pooled analysis, have demonstrated any advantage in feeding outcomes with HF support [14, 46, 49, 50]. Non-randomised studies suggest that oral feeds can be introduced in infants receiving HF [51, 52].

Studies in both the lab and clinical setting indicate that noise levels produced by HF are generally greater than, or similar to, those produced by CPAP [53, 54].

Studies that have more systematically assessed the response of parents and nursing staff have borne out initial reports that they find HF preferable to CPAP. Klingenberg et al. [47] reported that parents scored HF significantly higher in three domains: their child’s satisfaction, contact and interaction, and parental ability to take part in infant care. A single-centre survey of neonatal nurses revealed that they preferred HF to CPAP for infants of 28 weeks’ GA and above, believing it to be easier to set up and use, less likely to cause nasal trauma, more comfortable, and preferred by parents [55].

HF might also present the advantage of reduced cost, although there are currently few data to support this. A 2016 systematic review and economic evaluation performed by the National Institute for Health Research (UK), based on the results of four post-extubation RCTs, concluded that there was insufficient evidence to indicate whether HF treatment is cost-effective [56].

## Primary respiratory support

Prior to 2016, there was little published evidence from RCTs assessing the role of HF as primary respiratory support. Available data came from small studies, each including approximately 70 infants, [43, 46] and a subgroup of a larger trial that principally assessed post-extubation support. [14] Meta-analyses published in 2015 and 2016, including infants from these 4 studies, found no differences in treatment failure, intubation, or other important outcomes, and concluded that further adequately powered studies were required [10, 57]. The latter half of 2016 saw the publication of two larger studies, of differing methodology, designed to better evaluate primary HF.

A single-centre Italian study by Lavizzari et al. [49] randomised 316 preterm infants, of mean GA 33 weeks and birth weight 1.9 kg, to primary treatment with either HF, or CPAP/biphasic positive airway pressure (BiPAP). The primary outcome was treatment failure within 72 h, defined as the need for mechanical ventilation. The study allowed infants to receive intubation, surfactant treatment, and extubation (INSURE treatment), without regarding them as having treatment failure. The authors found similar rates of treatment failure (10.8% with HF and 9.5% with CPAP/BiPAP), with resultant risk difference (RD) 1.3% (one-sided 95% CI -6.0, 8.6), and concluded that HF was non-inferior to CPAP/BiPAP, relative to their specified non-inferiority margin of 10 percentage points. Despite the relatively low treatment failure rates, surfactant administration was common, occurring in >40% of infants in both treatment groups. Other outcomes did not differ significantly between treatment groups.

The authors acknowledged that their centre had relatively limited HF experience, and that treatment allocation could not be blinded. Other limitations included that the age at randomisation was not reported, and it was unclear if patients received respiratory support before randomisation took place. Furthermore, the trial was not prospectively registered in a clinical trials registry, and preliminary data from this trial had previously been published part way through recruitment, [58] so the authors were unblinded to the study outcomes, creating a risk of bias.

The HIPSTER trial, [13] led by our research group, included 564 infants of mean 32 weeks’ GA and 1.7 kg; infants had not received surfactant treatment and were median 1.4 h of age at randomisation. The study was designed to assess non-inferiority of HF, with a margin of 10%, but was stopped early on the advice of the independent safety monitoring committee due to a highly significant difference in the primary outcome: treatment failure within 72 h, defined by objective oxygenation, blood gas, apnoea and intubation criteria. Infants received surfactant treatment only after meeting treatment failure criteria.

Treatment failure occurred significantly more frequently in the HF infants (25.5% vs. 13.3%; RD 12.3%, 95% CI 5.8 to 18.7), but infants with HF failure could receive ‘rescue’ CPAP prior to intubation, and the resulting intubation rates were similar in the two treatment groups (15.5% vs. 11.5%; RD 3.9%, 95% CI -1.7 to 9.6). Again, blinding of the two treatments was not possible, and just over half of infants received a brief period of CPAP (median 1.5 h) prior to randomisation, which could have influenced outcomes.

Recently, another RCT comparing HF and CPAP as primary support in a single Korean unit was published [59]. This study included 85 of 87 randomised infants, mean 33 weeks’ GA and 2.0 kg, in a per-protocol analysis. Objective treatment failure criteria (respiratory acidosis, apnoea or hypoxia) were defined. Infants in both groups were permitted to receive nCPAP or bilevel CPAP at clinician discretion, prior to mechanical ventilation. Reported rates of treatment failure were 38.1% with HF compared to 20.9% with CPAP (RD 17.2%, 95% CI -1.9 to 36.2).

The higher rate of treatment failure seen in HF infants may be a reflection of the more consistent and slightly higher distending pressure produced by CPAP [16, 21]. This is likely to be beneficial in infants with acute RDS, and may explain the difference in risk of treatment failure seen with HF in HIPSTER, and the findings of the Lavizzari and post-extubation studies, in which many infants had received surfactant prior to determination of the primary outcome.

**Fig. 2:**
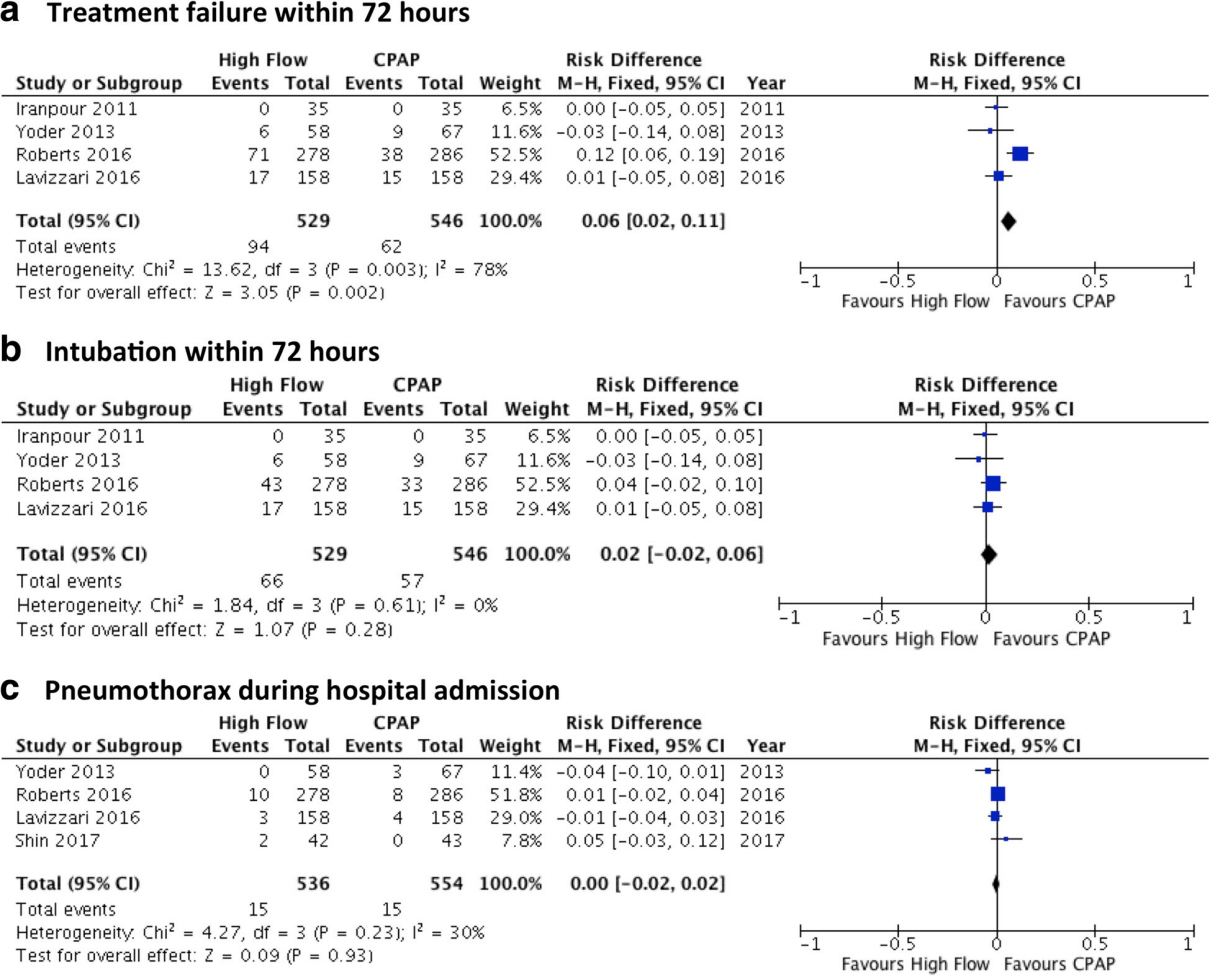
Pooled analyses of published randomised trials comparing High Flow and CPAP as primary support for preterm infants, for the outcomes: **a**. Treatment failure within 72 h; **b**. Intubation within 72 h; **c**. Pneumothorax during hospital admission

There are now >900 additional infants randomised in trials of primary HF support since the Cochrane Review was published in early 2016. An updated pooled analysis of key outcomes from published RCTs comparing HF and CPAP is shown in Fig. 2. Ultimately, whether clinicians choose to apply HF as primary support for preterm infants will depend on several factors. Those who place a high value on HF’s advantages (e.g. in units with high rates of nasal trauma associated with CPAP) may choose to use HF frequently, with the option of ‘rescue’ CPAP in those infants with HF failure. Those who have low rates of nasal trauma, or deal with populations with more severe RDS (e.g. those with low rates of antenatal steroid exposure) may prefer to use primary CPAP. Strategies to prospectively identify the infants most likely to be successfully treated with HF may facilitate wider use in this clinical setting. These studies have all included infants of 28 weeks’ GA or higher, and primary HF use in infants of lower gestations is not recommended based on current evidence.

## Post-extubation support

Early RCTs of post-extubation HF published between 2006 and 2010 (whether comparing HF and CPAP, or different HF devices) included relatively small study samples (≤40 infants), and low gas flows in comparison to current practice [44, 60, 61]. In 2013, the publication of three larger RCTs significantly added to the evidence base for HF use. The first included 132 very preterm infants (<32 weeks’ GA), who were randomised to either HF or CPAP at extubation [11]. Rates of extubation failure did not differ significantly (22% in the HF group and 34% in the CPAP group), nor did rates of reintubation (17% versus 24% respectively).

A similar study compared HF and CPAP using a non-inferiority design, designating a RD for treatment failure of 20 percentage points as the margin of non-inferiority for HF treatment [12]. This trial included 303 very preterm infants, and treatment failure (defined by pre-specified criteria) occurred in 34.2% of HF infants and 25.8% of CPAP infants; the RD for treatment failure with HF was 8.4% (95% CI -1.9, 18.7), thereby meeting the defined non-inferiority margin. This study also included ‘rescue’ CPAP treatment for those infants with HF failure, with the result that approximately half of these infants avoided reintubation within 7 days: 17.8%, versus 25.2% of CPAP infants. In those infants <26 weeks’ GA, the risk of HF failure was particularly high (81%) compared with 61% for CPAP. Although the 63 infants <26 weeks’ GA represent a small sample, given the high rate of HF failure and 20 percentage point increase compared with CPAP, the authors advised caution with HF use in this group.

The third RCT included infants between 28 and 42 weeks’ GA at birth. [14] Of the 432 infants included, 291 were randomised to HF or CPAP at extubation, and the primary outcome of reintubation within 72 h did not differ significantly between groups: 11.6% and 6.5% respectively.

A Cochrane Review, [10] updated in 2016, identified 6 published studies including 934 infants, who were randomised to either HF or CPAP as post-extubation support. Meta-analysis demonstrated that infants treated with HF were at no additional risk of treatment failure (typical risk ratio [RR] 1.21, 95% CI 0.95 to 1.55), reintubation (RR 0.91, 95% CI 0.68 to 1.20), or other adverse outcomes including death or BPD. The Cochrane Review authors noted that RCTs thus far have included relatively few extremely preterm infants (<28 weeks’ GA). Within the subgroup of infants from 28 to 32 weeks’ GA, HF resulted in a RR (95% CI) of 0.80 (0.44, 1.44) for treatment failure, and 0.51 (0.27, 0.97) for intubation in comparison to CPAP, suggesting that post-extubation HF (with the availability of rescue CPAP) may be a particularly appropriate strategy in this group of infants.

Three studies published since the Cochrane Review have included similar populations and methods to those previously published, and results were consistent with the previous Cochrane conclusions. Two studies showed similar rates of treatment failure and intubation with HF and CPAP, [62, 63] and the third small study found a higher rate of intubation in HF infants, although outcomes were reported for only half of the 108 infants randomised [64].

The available data seem to support the use of HF as an alternative to CPAP as post-extubation support for infants ≥28 weeks’ GA, but it is advised that ‘rescue’ CPAP, which prevented intubation of infants with HF failure in several studies, is available. Routine use of post-extubation HF in infants <28 weeks’ GA should be approached with caution.

## Weaning from CPAP

HF may also be used as a ‘weaning’ mode, to transition infants off CPAP support. Randomised trials assessing this approach have included far fewer infants than those assessing primary and post-extubation support, and have produced conflicting outcomes. One study found HF use to be associated with a significant increase in duration of oxygen therapy, and of respiratory support, in comparison to weaning directly from CPAP, [65] another found HF infants to have a significantly shorter duration of oxygen treatment and of hospital stay, [66] and other studies reported similar outcomes with either approach [67, 68]. There is no convincing evidence that use of HF in this manner is beneficial in weaning infants from CPAP, although given its advantages, it may be viewed as an alternative to CPAP in those infants who continue to require non-invasive support.

## Safety and later outcomes of high flow

The first major safety concern associated with HF therapy was an outbreak of infection with the Gram-negative species *Ralstonia,* resulting from contaminated humidifier cartridges in the Vapotherm 2000i device in 2005 [69]. Following the introduction of stricter infection control guidance by the manufacturer, there have been no further infection concerns since.

A further area of concern for clinicians, in keeping with the potential to generate high airway pressures discussed above, was the risk of over-distension and pulmonary air leak. Case reports of infants with complications including pneumothorax, pneumomediastinum, subcutaneous scalp emphysema, pneumocephalus and pneumo-orbitis gave some weight to these concerns [70–72]. Fortunately, available data from randomised trials have been reassuring, with no evidence of an increased pneumothorax risk with HF. The Cochrane Review in fact found a small reduction in the rate of pneumothorax (typical RD −0.02, 95% CI −0.03 to −0.00) in a pooled analysis of 896 infants from post-extubation studies. [10] Recent primary support trials have also reported similar rates of pneumothorax with HF and CPAP; a pooled analysis of these results is shown in Fig. 2 [13, 14, 49, 59].

Several RCTs have reported no increase in duration of support with HF, [11, 12, 49, 59] and in those that have, the difference has usually been small (1–2 days). [13, 14, 46] There is no evidence from RCTs to suggest that HF treatment is associated with an increased risk of BPD or death, either as individual or combined outcomes. Nor have these studies shown an increase in duration of hospitalisation, or other important clinical outcomes [10–14, 43, 49, 59].

Recent publications based on non-randomised data have raised concerns about the effect of HF use on respiratory outcomes, [73, 74] in particular prolonged respiratory support and BPD. However, limitations such as differing demographics in infants treated with HF, inconsistencies in the definition of BPD, and changes in practice during the study period, mean that these data do not allow firm conclusions to be drawn. HF is commonly applied after mechanical ventilation and/or CPAP, [75, 76] and it is conceivable that the infants receiving HF in these studies were those with the most significant lung disease, who required the most prolonged respiratory support. However, there are relatively few extremely preterm infants included in RCTs, and further research in this population is required. Exposure to prolonged respiratory support may be influenced in part by the approach to weaning HF applied by clinicians, and care should be taken to avoid unnecessarily prolonging support when weaning or cessation of treatment may be possible.

## Use in neonatal units

HF devices were incorporated into neonatal care before good quality RCT data were available to guide their use. The Vermont Oxford Network reported that HF use in infants born between 501 and 1500 g increased from 45% to 58% between 2006 and 2009 [77]. Surveys conducted between 2009 and 2011 showed HF to be in use in the majority of UK and Australasian NICUs, with the majority using gas flows of ≤8 L/min [40, 42, 78]. Concerningly in one UK survey, half of units stated that they selected the cannulae size that best fit the nostrils, rather than allowing a gas ‘leak’ around the prongs, which is an important safety mechanism. Many units also had no written HF guideline or policy in place [42]. Common reasons for HF use in Australian and UK units were as an alternative to CPAP, to wean from CPAP, and as post-extubation support [40].

Data reported from the 56 neonatal units in The Australian and New Zealand Neonatal Network (ANZNN) show that HF use increased from 8% of NICU registrants in 2009, to 27% in 2014. [79] HF use was most common in infants born at <28 weeks’ GA. Amongst all very preterm infants from 2009 to 2012, HF was commenced at a median 17 days of age, and the majority of infants had received CPAP (90%), endotracheal ventilation (71%), or both (64%) prior to HF, with primary HF use being rare (2% of infants) [75].

More recent publications indicate further growth in HF use in the UK, [76] use in the majority of Japanese NICUs, [80] and the introduction of HF use during neonatal inter-hospital transport [81].

## Future research directions

There remain several areas of HF practice in which further evidence is needed. Given the concerns raised about prolonged exposure to respiratory support, studies assessing the best approach to weaning and ceasing HF should be conducted. Extremely preterm infants, who are those at greatest risk of BPD, should be further studied, to ensure that HF use is not associated with important adverse outcomes.

Studies thus far have evaluated HF at the gas flows suggested by device manufacturers, which are currently a maximum of 8 L/min in the neonatal population. Given that evidence suggests adverse events (particularly pneumothorax) at these flows are rare, there is the possibility to study the use of higher gas flows, which may produce a greater distending pressure, potentially beneficial in conditions such as RDS. Some clinicians continue to report an anecdotal preference for one HF device over another and, with the exception of a single underpowered study, [61] there has been little research in this area. HF may be of value as a method of stabilisation in the delivery room, as reported in a small observational study of infants <30 weeks’ GA at birth, [82] but this has yet to be assessed in a randomised trial.

The available RCT evidence for neonatal HF use relates exclusively to NICUs, but HF use in non-tertiary neonatal units is commonly reported, and increasing. A multi-centre RCT comparing HF and CPAP in Australian non-tertiary neonatal units is currently underway (ACTRN12614001203640).

## Conclusions

High Flow is now well established as an important part of neonatal respiratory care, and has a number of potential benefits for preterm infants. While it appears that HF can be applied successfully as primary support for many infants >28 weeks’ GA, it is less effective than CPAP in preventing treatment failure, and factors such as severity of RDS in the treated population, and approach to surfactant should be considered. The evidence suggests that post-extubation HF is suitable for infants >28 weeks’ GA. The availability of rescue CPAP is an important part of the treatment pathway in both primary and post-extubation use, ensuring infants treated with HF are not at increased risk of intubation. Research into the optimal approach to weaning HF, and its use in extremely preterm infants, is required to ensure it is applied in such a way that produces maximal benefit for infants and their families, whilst avoiding unintended adverse effects.
